# Cost-Effectiveness of an Interdisciplinary, Internet-Based Transgender Health Care Program in Germany: Economic Evaluation Alongside a Randomized Controlled Trial

**DOI:** 10.2196/66371

**Published:** 2025-06-19

**Authors:** Thomas Grochtdreis, Hans-Helmut König, Janis Renner, Susanne Sehner, Arne Dekker, Peer Briken, Timo O Nieder, Judith Dams

**Affiliations:** 1 Department of Health Economics and Health Services Research, Hamburg Center for Health Economics, University Medical Center Hamburg-Eppendorf, Martinistraße 52, Hamburg, 20251, Germany, 49 40741053382, 49 40741040261; 2 Institute for Sex Research, Sexual Medicine and Forensic Psychiatry, University Medical Center Hamburg-Eppendorf, Hamburg, Germany; 3 Institute of Medical Biometry and Epidemiology, University Medical Center Hamburg-Eppendorf, Hamburg, Germany

**Keywords:** cost-effectiveness analysis, quality-adjusted life years, transgender, gender diverse, gender incongruence, mental health, gender dysphoria, telehealth, interdisciplinary, internet-based, genderqueer, gender nonconforming people, health care program, Germany, interdisciplinary care, gender diversity, eHealth, health care services

## Abstract

**Background:**

The provision of specialized, professionally coordinated, and interdisciplinary care is relevant for the care of transgender and gender diverse (TGD) people diagnosed with gender incongruence or gender dysphoria. In remote areas outside the metropolitan regions, however, transgender health care structures are rarely adequate or within reach. In order to improve transgender health care for TGD people, an interdisciplinary, internet-based transgender health care program (i²TransHealth) has been developed.

**Objective:**

The aim of this study was to determine the cost-effectiveness of i²TransHealth for TGD people from remote areas with no or insufficient transgender health care structures either exploring their gender identity or being in an early phase of transition from a societal perspective.

**Methods:**

This study was conducted alongside a randomized controlled trial comparing the effectiveness of i²TransHealth with a waiting list. The i²TransHealth intervention consisted of a telehealth-based eHealth intervention including one-to-one chat conversations with study therapists in combination with office-based regular care provided by general physicians and psychiatrists when needed. As health effect measures, quality-adjusted life years (QALYs) were calculated based on the EuroQol 5-dimension 5-level index, and reliable improvement on the Global Severity Index of the Brief Symptom Inventory-18 (BSI-18 GSI) was used. Health care service usage was assessed using service receipt inventories. The cost-effectiveness of i²TransHealth compared with a waiting list was assessed using the adjusted incremental cost-effectiveness ratio (ICER) based on seemingly unrelated regressions. Furthermore, the uncertainty of the ICER was assessed using cost-effectiveness planes and cost-effectiveness acceptability curves.

**Results:**

Participants in the intervention group (IG; n=88) and the control group (CG; n=80) were on average aged 26 and 27 years, respectively. The mean QALYs of participants in the IG and CG were both 0.28 (SE 0.00) during the 4-month follow-up period. With 23.02%, participants in the IG had statistically significantly higher reliable improvement on the BSI-18 GSI compared with participants in the CG (9.21%, *P*=.01). The mean 4-month total costs were statistically significantly higher among the participants in the IG (+€1390, *P*=.002; a currency exchange rate of €1=US $1.14 was applicable as of December 31, 2020). The corresponding ICER of i²TransHealth was €254,021 per additional QALY, and €10,786 per additional reliable improvement on the BSI-18 GSI, respectively. The corresponding probability of cost-effectiveness of i²TransHealth was 20% at a willingness-to-pay (WTP) of €150,000 per additional QALY and 75% at a WTP of €15,000 per additional reliable improvement on the BSI-18 GSI.

**Conclusions:**

From a societal perspective, i²TransHealth was unlikely to be cost-effective, even at high WTP per additional QALY. However, the comparison of i²TransHealth with a waiting list could have led to a distortion of the results with regard to health care service usage. When considering additional reliable improvement on the BSI-18 GSI as health effect measure, the probability of cost-effectiveness of i²TransHealth is unclear depending on the WTP.

## Introduction

Transgender and gender diverse (TGD) people, among others, are individuals “whose gender identity or expression differs from the gender socially attributed to their assigned sex at birth” [1]. According to the *International Classification of Diseases, 11th Revision* (*ICD-11*) and the *Diagnostic and Statistical Manual of Mental Disorders, Fifth Edition* (*DSM-5*), TGD people either exploring their gender identity or being in an early phase of transition can be clinically diagnosed with gender incongruence (GIC) or gender dysphoria (GD) [2,3]. The needs of TGD people either exploring their gender identity or being in an early phase of transition are very heterogeneous, which is why transgender health care primarily aims at leading a self-determined and authentic life aligned with one’s gender identity, improving the (health-related) quality of life and reducing the psychological distress associated with GIC or GD [4-6]. Access to specialized transgender health care, however, is limited for TGD people in Germany, especially outside metropolitan areas [7,8].

In order to overcome this issue and to direct TGD people through the decision-making process with respect to transgender health care, there is a need for coordinated and integrated transgender health care. In northern Germany, the Outpatient Unit for Sexual Health and Transgender Care at the Institute for Sex Research and Sexual Medicine, University Medical Center Hamburg-Eppendorf (UKE) is the only center specialized in transgender health care. However, in remote areas outside the UKE catchment area, the problem of lack of access to transgender health care remains.

One way to address this lack of access to transgender health care is through eHealth interventions and office-based regular care provided by general physicians and psychiatrists who are sensitized to transgender health care [9]. It has already been demonstrated that establishing eHealth interventions and TGD-informed office-based regular care provided by general physicians and psychiatrists are approaches to reduce physicians’ reservations and to guide TGD people through transgender health care in remote areas [7,8,10-14]. In order to improve transgender health care, an interdisciplinary, internet-based transgender health care (i²TransHealth) program was developed [4,6]. The program consists of an eHealth intervention targeting TGD people from remote areas either exploring their gender identity or being in an early phase of transition. Furthermore, the program consists of training a network of office-based regular care provided by general physicians and psychiatrists when needed in remote areas outside the catchment area of the UKE with the aim of complementing the eHealth intervention with the provision of TGD-informed care. In this regard, the effectiveness analysis showed that i²TransHealth was able to prevent deterioration in psychological distress and health-related quality of life compared with a waiting list [6,8].

To our knowledge, the cost-effectiveness of interdisciplinary, internet-based transgender health care programs has not yet been evaluated. The focus of the existing cost-effectiveness analyses in TGD people was on HIV prevention in transgender women in Brazil, Peru, and Thailand [15-19]. One further analysis was on the cost-effectiveness of health insurance coverage for medically necessary and preventive services compared with no coverage in the US adult transgender population [20]. The effectiveness of interventions related to access to transgender health care has rarely been analyzed and not at all in Germany [21-24]. Thereby, those interventions aimed at reducing identity-related stigma and mental health issues, and minority stress, suicide prevention, and increasing treatment-seeking intentions.

Therefore, in order to contribute to research related to access to transgender health care, the aim of this study was to evaluate the cost-effectiveness of the i²TransHealth interdisciplinary, internet-based transgender health care program compared with a waiting list for TGD people from remote areas with no or insufficient transgender health care structures either exploring their gender identity or being in an early phase of transition from a societal perspective (SP). It was hypothesized that i²TransHealth leads to a reduction in direct health care costs as well as to less absenteeism from work and reduced productivity at work. Furthermore, it was hypothesized that i²TransHealth leads to an increased number of quality-adjusted life years (QALYs).

## Methods

### Sample

This study was part of a single-blind randomized controlled trial to compare the effectiveness of i²TransHealth with a waiting list for TGD people from remote areas either exploring their gender identity or being in an early phase of transition in northern German primary care (trial registration NCT04290286) [4,6]. Recruitment of TGD people took place by direct contact of the Outpatient Unit for Sexual Health and Transgender Care at the Institute for Sex Research and Sexual Medicine by phone, via the trial website [25], or through a network of local general physicians and psychiatrists established and trained specifically for the study. Furthermore, recruitment took place through TGD-related organizations in the participating federal states, social media, and publications in German journals for medical and mental health professionals. Hereby, information about i²TransHealth was provided and an appointment was made for an initial face-to-face interview. The study setting for the recruitment was TGD people with GIC or GD who lived at least 50 km outside of Hamburg in northern Germany.

Inclusion criteria of the study were a clinical diagnosis of either GIC (according to the *ICD-11* [2]) or GD (according to the *DSM-5* [3]) based on the initial face-to-face interview, being 18 years or older, and living at least 50 km outside Hamburg in the federal state Bremen, Mecklenburg-Western Pomerania, Lower Saxony, or Schleswig-Holstein. Furthermore, the people had to be cognitively, verbally, and auditorily able to use the video consultation system and did not have already started transition-related treatments elsewhere. People with an indication for inpatient psychiatric treatment, with suicidal tendencies, with intellectual disorder of development, or with an acute addictive drug intoxication were excluded. Furthermore, people without technical requirements for the video consultations or with insufficient knowledge of German or English language were excluded.

After informed consent, people were included in the study as participants and randomly assigned to the intervention group (IG) or the control group (CG). Assessment took place at baseline (T0) and 4 months after baseline (T1). A detailed description of the i²TransHealth trial can be found in studies by Nieder et al [4,6].

### Ethical Considerations

All procedures performed in studies involving human participants were in accordance with the ethical standards of the institutional and national research committees and with the 1964 Declaration of Helsinki and its later amendments or comparable ethical standards. The Ethical Review Board of the Hamburg Medical Association has given ethical approval (PV7131). Written informed consent was given after an initial on-site interview to clarify participation requirements and implementation of the video consultation before study inclusion. The participants received no financial compensation for their participation in the study. All data used in this study were anonymized and deidentified. No identification of individual participants in any images in this paper or Multimedia Appendices is possible. The Consolidated Health Economic Evaluation Reporting Standards 2022 (CHEERS 2022) statement for economic evaluations was followed (Checklist 1) [26].

### Interventions

Participants in the IG received an eHealth intervention via an eHealth platform according to the i²TransHealth interdisciplinary, internet-based transgender health care program [25]. Clinical interventions took place via video consultation every 2 weeks. Furthermore, participants in the IG had the opportunity to contact study therapists via messaging in the eHealth platform, who responded within 48 hours on weekdays. After the end of treatment, participants in the IG were transferred to office-based regular coordinated and integrated transgender health care provided by psychiatrists of the Outpatient Unit for Sexual Health and Transgender Care.

Participants in the CG were placed on a waiting list for 4 months until they were offered either an eHealth intervention according to the i²TransHealth interdisciplinary, internet-based transgender health care program or office-based regular coordinated and integrated transgender health care.

### Measures

As primary health outcome measure for the cost-effectiveness analysis, the EuroQol 5-dimension 5-level (EQ-5D-5L) was used to measure generic health-related quality of life (HrQoL) [27]. The EQ-5D-5L measures HrQoL on the 5 dimensions: mobility, self-care, usual activities, pain or discomfort, and anxiety or depression using 5 questions. Each answer to the respective question has 5 ordinal levels ranging from “no problems” to “extreme problems.” Based on the descriptive system of the EQ-5D-5L, which is able to describe 3125 health states, an EQ-5D-5L index can be calculated. By assigning societal preferences from the German general population to the EQ-5D-5L health states, the EQ-5D-5L index maps HrQoL on a scale ranging from −0.661 (extreme problems in all 5 dimensions) to 1 (no problems in any dimension) [28].

As alternative measures, subjective HrQoL was measured using the visual analog scale of the EuroQol 5-dimension 5-level (EQ-VAS) [27]. The EQ-VAS maps subjective HrQoL on a scale ranging from 0 (worst imaginable health state) to 100 (best imaginable health state). Furthermore, as secondary health outcome measure, the Global Severity Index of the Brief Symptom Inventory (BSI-18 GSI) was used to measure the psychological distress with respect to somatization, anxiety, and depression [29]. The BSI-18 GSI score ranges from 0 to 72 [30].

For the assessment of health care services usage, adapted versions of the German Client Socio-Demographic and Service Receipt Inventory and the Questionnaire for the Assessment of Medical and Non-Medical Resource Utilization in Mental Disorders were used [27,31]. Furthermore, participants were asked about their absenteeism from work as well as their productivity at work. In order to assess productivity at work, overall work performance was rated on a scale ranging from 0 to 10 [32].

Participants were also asked about their age, sex assigned at birth, gender identity, relationship status, school-leaving qualification, professional education, education status, occupational status, federal state of residence, and health insurance. Thereby, the self-declared gender identity was trichotomized in trans man or trans masculine, trans woman or trans feminine, and nonbinary gender. Furthermore, self-reported somatic and psychiatric morbidities were assessed at baseline.

### Calculation of Health Effects

QALYs were calculated by the EQ-5D-5L index and the time spent in the respective health state [27,28]. Thereby, the EQ-5D-5L index values at T0 and T1 were linearly interpolated and multiplied with 4 divided by 12. Accordingly, the maximum and minimum possible QALYs during the 4 months after baseline were 0.33 and −0.22, respectively. Furthermore, the EQ-VAS score was used to calculate QALYs. For this, the EQ-VAS scores were divided by 100 and QALYs were calculated in the same way as described above.

As secondary health effect measure, a reliable improvement on the BSI-18 GSI was derived from the effectiveness analysis of i²TransHealth, which used the BSI-18 GSI as the primary outcome measure [6]. A reduction of ≥5.46 of the BSI-18 GSI score between T0 and T1 was defined as reliable improvement on the BSI-18 GSI, based on the reliable change index calculated with reference values of the BSI-18 GSI of the reference cohort (mean 4.66, SD 7.44, Cronbach α 0.93) [6,30,33]. Reliable improvement on the BSI-18 GSI was preferred over point value change because willingness-to-pay (WTP) per additional reliable improvement on the BSI-18 GSI can be better estimated than per additional point value change of the BSI-18 GSI to interpret the cost-effectiveness acceptability curve.

### Calculation of Costs

For the calculation of costs of health care services usage, quantities of usage were monetarily valuated by standardized unit costs for the German health care system [34-36]. The German consumer price index was used to inflate the standardized unit costs to 2020 price levels [37]. A currency exchange rate of €1=US $1.14 was applicable as of December 31, 2020. Medication use costs were monetarily valuated by prices per pack based on the German pharmaceutical database “Rote Liste” from the year 2020 [38]. For informal care, it was assumed that it could have been substituted by formal care [34]. Thus, usage of informal and formal care was monetarily valuated using the gross hourly wage of people in the commercial sector “social care for older adults and disabled people” based on the gross labor cost database from the Federal Statistical Office of Germany [39]. Absenteeism from work and reduced productivity at work were converted into lost working hours assuming an 8-hour working day and monetarily valuated using the gross hourly wage of people in manufacturing and services sectors, pursuing the human capital approach (indirect costs) [39]. Thereby, reduced productivity at work (*a*) was calculated as *a*=(1–*w*/10)*×d* , where *w* is the overall work performance and *d* is the days at work.

The costs of the eHealth intervention consisted of the costs of usage of psychotherapists who carried out clinical interventions via video consultation every 2 weeks and responded to the messaging on the eHealth platform. The usage of psychotherapists was monetarily valuated by standardized unit costs [34]. The costs of the eHealth intervention were added to the total costs 4 months after baseline of the participants in the IG.

### Statistical Analysis

The primary data analysis was based on the analysis population of the effectiveness analysis of i²TransHealth (n=168) [6]. Of the total intention-to-treat population, which includes all randomized participants (n=174), 6 study participants who were lost to postassessment (no response for the primary outcome of the effectiveness analysis, BSI-18 GSI) were not included in the primary data analysis. As missing information on the 144 included variables varied between 0% (n=0) and 2.8% (n=4) and the overall missing percentage was 0.5% (117 of 24,192 records), missing values were not imputed. According to van Buuren [40], in that case, a complete case analysis produces correct standard errors and significance levels compared with an analysis of data without missing information, yet under the assumption that the data are missing completely at random.

The unadjusted total costs and health effects up to 6 months before baseline and up to 4 months after baseline were compared using *F* tests based on linear regression.

The costs and health effects up to 4 months after baseline were further compared using seemingly unrelated regressions with robust standard errors based on nonparametric, bias-corrected, and accelerated bootstrapping with 1000 replications [41]. Seemingly unrelated regression models are joint estimates from 2 or more regression models with separate error terms, yet correlated errors associated with the dependent variables. Thereby, in the regression models with costs as dependent variable, gender identity, age, and costs up to 6 months before baseline were added as independent variables. In the regression models with health effects as dependent variable, the independent variables gender identity, age, EQ-5D-5L index and BSI-18 GSI at baseline were added.

The results from the seemingly unrelated regression models were used to calculate adjusted incremental cost-effectiveness ratios (ICERs), that is, the difference in mean total costs within 4 months after baseline divided by the difference in mean health effects between the services users in the IG and CG. Furthermore, cost-effectiveness planes based on bootstrapping the results of the seemingly unrelated regression models were used to assess the uncertainty around the ICERs [42]. In order to provide probabilities of cost-effectiveness for different WTP thresholds per additional health effect, the uncertainty around the ICERs was furthermore assessed using cost-effectiveness acceptability curves based on the net-benefit approach [43,44]. Thereby, the probability of cost-effectiveness is defined as the proportion of bootstrapped ICERs that falls below the respective WTP threshold per additional health effect.

All data analyses were performed using Stata/MP 18.0 (StataCorp). All statistics were tested 2-sided with a significance level of .05. All *P* values were considered as descriptive only.

### Additional Analyses

Subgroup analyses were conducted for trans men, trans women, and nonbinary people. Analyses were repeated from a health care payer’s perspective (PP) by excluding costs due to absenteeism from work and reduced productivity. Analyses were also repeated with QALYs calculated using the EQ-VAS as health effect.

Furthermore, the analyses were repeated with a per-protocol population (without 7 study participants who were excluded due to violation of inclusion criteria or due to discontinued condition). The analyses were also repeated with multiply imputed data (multiple imputation by chained equations with predictive mean matching; m=20 imputations), as despite a low overall missing percentage, the total number of persons with missing information was not negligible (n=18, 10.7%). The analyses were also repeated with costs winsorized at the 95th percentile, with high intervention costs and with only mental health–related costs to check the robustness of the assumptions in the calculation of intervention costs. For the calculation of high intervention costs, the usage of psychotherapists was monetarily valuated by labor costs calculated based on the collective agreement for employees in the public sector for German federal states [45] instead on standardized unit costs [34].

## Results

### Sample Characteristics

**Figure 1.**
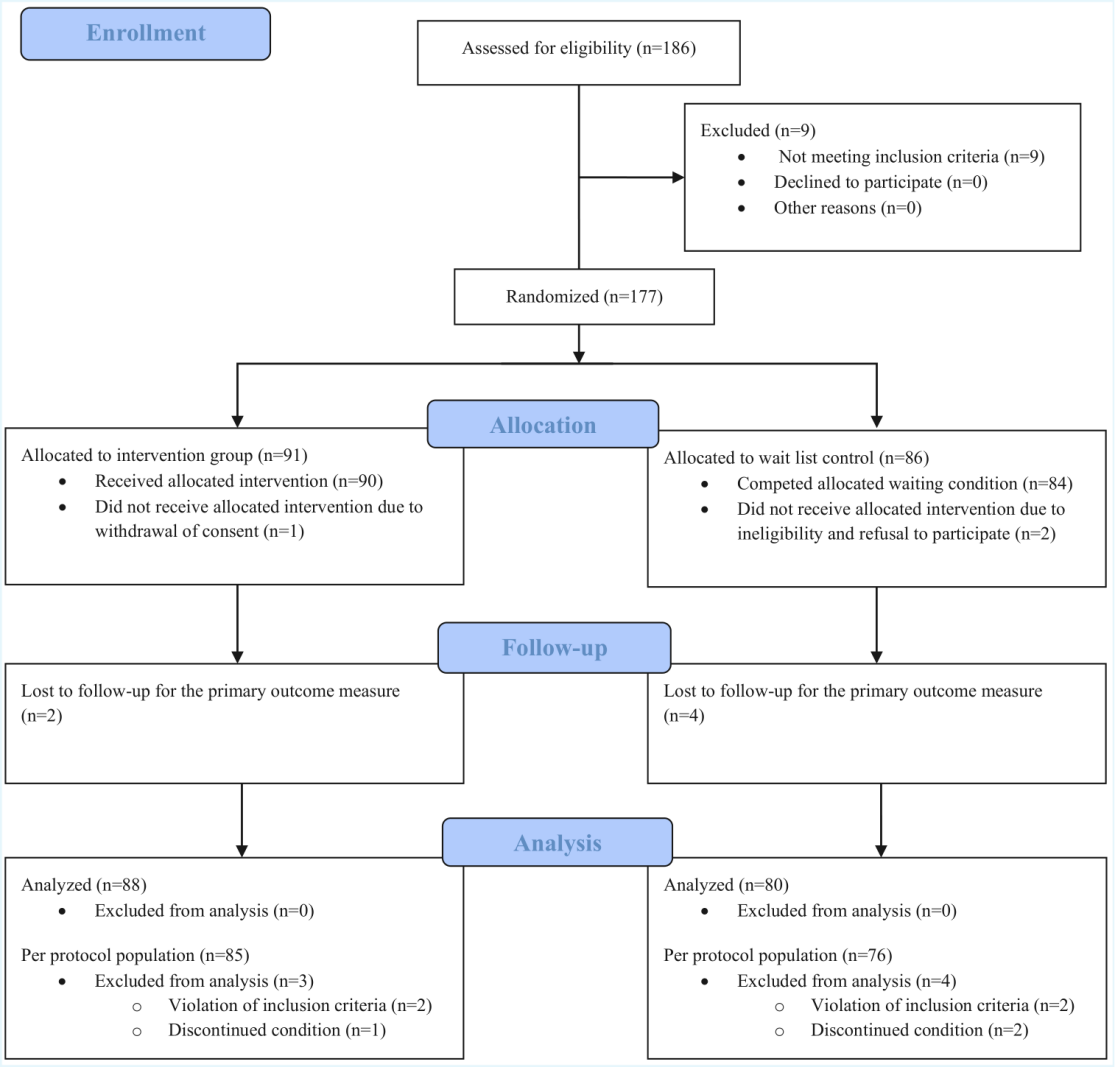
Consolidated Standards of Reporting Trials (CONSORT 2010) flow diagram of the study of the i²TransHealth interdisciplinary, internet-based transgender health care program compared with a waiting list for transgender and gender diverse people in northern Germany.

The participant flow and reasons for dropout throughout the study are presented in Figure 1 and the sociodemographic characteristics of the sample are presented in Table 1. Services users in the IG (n=88) and the CG (n=80) were on average aged 26 and 27 years, respectively. The absolute majority of the sample was assigned with female sex at birth (n=50, 56.8% and n=43, 53.8%). Among participants in the IG and CG, 47.7% (n=42) and 38.8% (n=31) identified as trans masculine, 34.1% (n=30) and 33.8% (n=27) as trans feminine, and 18.2% (n=16) and 27.5% (n=22) as nonbinary, respectively. The majority of the participants in the IG and CG completed school with less than a vocational diploma (n=56, 63.6% and n=43, 53.8%). On average, participants in the IG and CG had 1 somatic and psychiatric morbidity, respectively (Table 1).

**Table 1.** Sociodemographic and clinic characteristics of the study sample of the i²TransHealth interdisciplinary, internet-based transgender health care program compared with a waiting list for transgender and gender diverse people in northern Germany at T0 (N=168).

Sociodemographic and clinic characteristic	Intervention group (n=88)	Control group (n=80)	*P* value^a^
Age (y), mean (SD)	26.4 (8.6)	27.3 (11.3)	.41
Female sex assigned at birth, n (%)	50 (56.8)	43 (53.8)	.92
Gender identity, n (%)			.31
Trans man or trans masculine	42 (47.7)	31 (38.8)	
Trans woman or trans feminine	30 (34.1)	27 (33.8)	
Nonbinary	16 (18.2)	22 (27.5)	
Relationship status, n (%)			.89
Single	57 (64.8)	51 (63.8)	
In a relationship or married	31 (35.2)	29 (36.3)	
Education, n (%)			.13
Less than vocational diploma	56 (63.6)	43 (53.8)	
Vocational diploma or academic secondary diploma	32 (36.4)	37 (46.3)	
Employment, n/N (%)			.86
Employed or self-employed	38/79 (48.1)	35/71 (49.3)	
In training (school, university, and vocational training)	41/79 (51.9)	36/71 (50.7)	
“I cannot or do not want to answer”	9/88 (10.2)	9/80 (11.3)	
Federal state of residence, n (%)			.59
Bremen	5 (5.7)	3 (3.8)	
Mecklenburg-Western Pomerania	10 (11.4)	6 (7.5)	
Lower Saxony	49 (55.7)	49 (61.3)	
Schleswig-Holstein	24 (27.3)	22 (27.5)	
Health insurance, n (%)			.47
Statutory health insurance	71 (80.7)	65 (81.3)	
Statutory health insurance (plus private supplementary insurance)	13 (14.8)	8 (10)	
Private health insurance	2 (2.3)	5 (6.3)	
Number of morbidities, median (IQR)			
Somatic morbidities	1.0 (0.0-2.0)	1.0 (0.0-2.0)	.99
Psychiatric morbidities	1.0 (0.0-2.0)	1.0 (0.0-2.0)	.51

a Comparisons of categorical characteristics between participants of the intervention group and control group were analyzed using Pearson chi-square tests; comparisons of age and number of morbidities between participants of the intervention group and control group were analyzed using Wilcoxon rank-sum tests.

### Health Effects and Costs

The unadjusted mean EQ-5D-5L index score of the participants in the IG and CG was 0.83 (SE 0.02) and 0.87 (SE 0.02) at T0, respectively (Table 2). The difference between groups was not statistically significant (−0.04, SE 0.03, *P*=.09; Table 2). At T1, the EQ-5D-5L index score of the participants in the IG increased to 0.85 (SE 0.02), whereas the EQ-5D-5L index score of the participants in the CG decreased to 0.84 (SE 0.02). Again, the difference between groups was not statistically significant (0.01, SE 0.03; *P*=.71). The unadjusted mean EQ-VAS score was not statistically significantly different between participants in the IG and CG at any time point.

**Table 2.** Unadjusted mean intervention costs, total costs, and health effects of the study sample of the i²TransHealth interdisciplinary, internet-based transgender health care program compared with a waiting list for transgender and gender diverse people in northern Germany until 6 months before baseline (T0) and until 4 months after baseline (T1).

Cost category^a^ or measure of health effect	IG^b^	CG^c^	Difference IG−CG	*P* value
Intervention costs, mean (SE)				
T0-T1 (n=168)	€734 (€10)	€0 (€0)	€734 (€10)	<.001
Total costs (PP^d^), mean (SE)				
T0 (n=157)	€2856 (€762)	€2278 (€570)	€578 (€987)	.56
T1 (n=156)^e^	€2422 (€337)	€880 (€254)	€1542 (€429)	<.001
Total costs (SP^f^), mean (SE)				
T0 (n=157)	€3703 (€794)	€3383 (€648)	€319 (€1054)	.76
T1 (n=156)^e^	€2999 (€364)	€1494 (€266)	€1505 (€429)	.001
EQ-5D-5L^g^ index, mean (SE)				
T0 (n=168)	0.83 (0.02)	0.87 (0.02)	−0.04 (0.03)	.09
T1 (n=167)	0.85 (0.02)	0.84 (0.02)	0.01 (0.03)	.71
EQ-VAS^h^, mean (SE)				
T0 (n=168)	68.52 (20.56)	72.73 (23.27)	−4.20 (3.38)	.22
T1 (n=167)	70.07 (2.20)	70.45 (2.50)	−0.38 (3.32)	.91

a A currency exchange rate of €1=US $1.14 was applicable as of December 31, 2020.

b IG: intervention group.

c CG: control group.

d PP: health care payer’s perspective.

e Includes intervention costs.

f SP: societal perspective.

g EQ-5L-5L: EuroQol 5-dimension 5-level.

h EQ-VAS: visual analog scale of the EuroQol 5-dimension 5-level.

The unadjusted mean 6-month total costs from an SP of the participants in the IG and CG were €3703 (SE €794) and €3383 (SE €648) at T0, respectively. At T1, the unadjusted mean 4-month total costs of the participants in the IG and CG were €2999 (SE €364) and €1494 (SE 266), respectively. The difference in unadjusted mean total costs between the groups was statistically significant only at T1 (+€1505, SE €429; *P*=.001). The mean 4-month total costs at T1 of the participants in the IG included the mean intervention costs of €734 (SE €10).

The adjusted mean QALYs during the 4-month follow-up were 0.28 (SE 0.00) for both, the participants in the IG and CG (Table 3). The adjusted reliable improvement on the BSI-18 GSI, with a 13.8% higher reliable improvement on the BSI-18 GSI among service users in the IG (*P*=.01; IG 23.0%, CG 9.2%).

The adjusted mean 4-month total costs from SP were statistically significantly higher for participants in the IG than for services users in the CG (+€1390, *P*=.002; Table 3). Considering the individual cost categories, costs for medical counseling (+€280, SE €134; *P*=.04), and transportation (+€37, SE €16; *P*=.007) were statistically significantly different, with higher costs among participants of the IG. In all other categories, the differences between groups were not statistically significant.

**Table 3.** Adjusted means and differences (cost-differences adjusted for gender identity, age, and total costs at baseline, and QALY^a^ differences or differences in reliable improvement on the BSI-18 GSI^b^ adjusted for gender identity, age, EQ-5D-5L^c^ index, and BSI-18 GSI at baseline by seemingly unrelated regression) between services users of the IG^d^ and CG^e^ in mean costs (by cost category), QALYs and reliable improvement on the BSI-18 GSI of the i²TransHealth interdisciplinary, internet-based transgender health care program compared with a waiting list for transgender and gender diverse people in northern Germany during 4-month follow up (n=150).

Cost category^f^ or measure of health effect	IG (n=81), mean (SE)	CG (n=69), mean (SE)	Difference IG−CG^g^	*P* value
Inpatient care and rehabilitation	€405 (€158)	€275 (€171)	€131 (€234)	.58
Outpatient physician and mental health specialist services	€379 (€51)	€302 (€55)	€77 (€75)	.30
Outpatient nonphysician services	€48 (€24)	€59 (€26)	−€12 (€36)	.74
Medical aids	€30 (€14)	€28 (€15)	€2 (€21)	.91
Medical counseling	€314 (€91)	€34 (€98)	€280 (€134)	.04
Group therapy or individual therapy	€7 (€5)	€7 (€4)	−€0 (€16)	.95
Transportation	€56 (€11)	€18 (€12)	€37 (€16)	.02
Medication	€37 (€33)	€89 (€36)	−€52 (€49)	.30
Nursing care	€370 (€163)	€102 (€176)	€269 (€241)	.26
Indirect costs^h^	€542 (€96)	€618 (€104)	−€76 (€142)	.59
Total costs (PP^i^)^j^	€2381 (€280)	€915 (€303)	€1466 (€414)	<.001
Total costs (SP^k^)^j^	€2923 (€296)	€1533 (€321)	€1390 (€439)	.002
QALY (EQ-5D-5L)	0.28 (0.00)	0.28 (0.00)	0.01 (0.00)	.07
QALY (EQ-VAS^l^)	0.23 (0.01)	0.23 (0.01)	0.00 (0.01)	.93
Reliable improvement on the BSI-18 GSI^m^	23.0%	9.2%	13.8%	.01

a QALY: quality-adjusted life year.

b BSI-18 GSI: Global Severity Index of the Brief Symptom Inventory-18.

c EQ-5D-5L: EuroQol 5-dimension 5-level.

d IG: intervention group.

e CG: control group.

f A currency exchange rate of €1=US $1.14 was applicable as of December 31, 2020.

g The difference shown may not match the difference between intervention group and control group due to rounding.

h Includes costs from absenteeism from work and reduced productivity at work.

i PP: health care payer’s perspective.

j Includes intervention costs.

k SP: societal perspective.

l EQ-VAS: visual analog scale of the EuroQol 5-dimension 5-level.

m Data with regard to reliable improvement on the BSI-18 GSI were taken from the effectiveness analysis of i²TransHealth [6].

### Cost-Effectiveness of i²TransHealth

**Figure 2.**
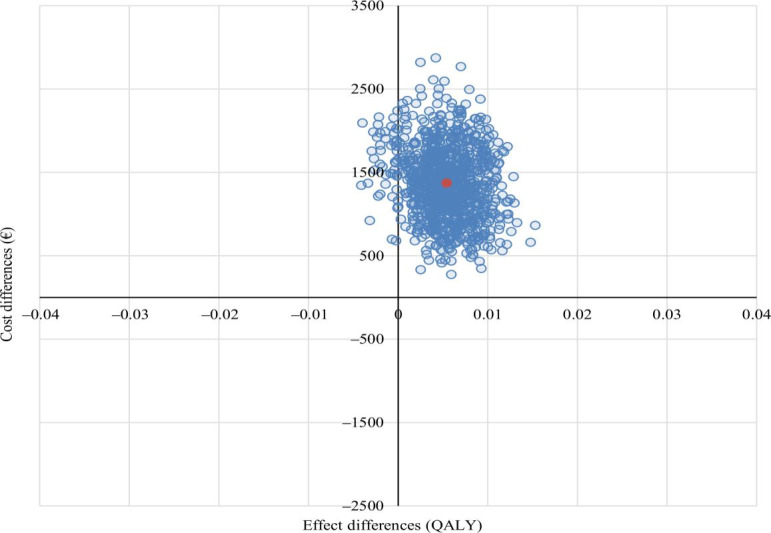
Adjusted cost-effectiveness plane of the i²TransHealth interdisciplinary, internet-based transgender health care program compared with a waiting list for transgender and gender diverse people in northern Germany: primary analysis from societal perspective with QALYs based on the EuroQol 5-dimension 5-level as health outcome (cost-differences adjusted for gender identity, age, and total costs at baseline, and effect differences adjusted for gender identity, age, EuroQol 5-dimension 5-level index, and Global Severity Index of the Brief Symptom Inventory-18 at baseline by seemingly unrelated regression with bootstrapped SEs). A currency exchange rate of €1=US $1.14 was applicable as of December 31, 2020. QALY: quality-adjusted life year.

**Figure 3.**
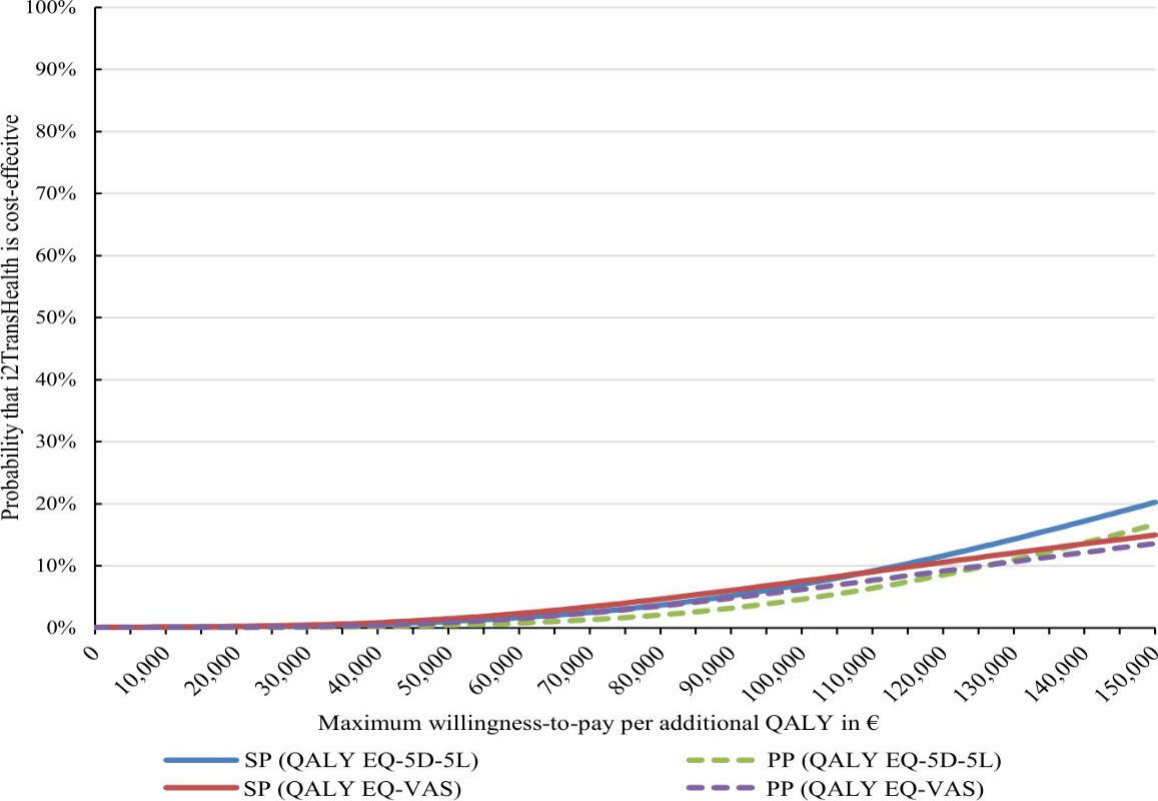
Adjusted cost-effectiveness acceptability curves for an additional QALY of the i²TransHealth interdisciplinary, internet-based transgender health care program compared with a waiting list for transgender and gender diverse people in northern Germany: primary analyses from a societal perspective and payer’s perspective with QALY based on the EQ-5D-5L as health outcome and additional analyses with QALY based on the EQ-VAS as health outcome (cost-differences adjusted for gender identity, age, and total costs at baseline, and effect differences adjusted for gender identity, age, EQ-5D-5L index, and Global Severity Index of the Brief Symptom Inventory-18 at baseline by seemingly unrelated regression with bootstrapped SEs). A currency exchange rate of €1=US $1.14 was applicable as of December 31, 2020. EQ-5D-5L: EuroQol 5-dimension 5-level; PP: health care payer’s perspective; QALY: quality-adjusted life year; SP: societal perspective; EQ-VAS: visual analog scale of the EuroQol 5-dimension 5-level..

From an SP, the ICER of i²TransHealth was €239,118 per additional QALY. With 99% of the bootstrapped ICERs being in the northeastern quadrant, the cost-effectiveness plane indicated that i²TransHealth was highly likely to be associated with higher costs and QALYs compared with the waiting list (Figure 2). The probability of cost-effectiveness was 0% at a WTP of €0 per additional QALY. At a WTP of €50,000 per additional QALY, the probability of cost-effectiveness was 1%, yet at a WTP of €150,000, the probability increased to 20% (Figure 3).

**Figure 4.**
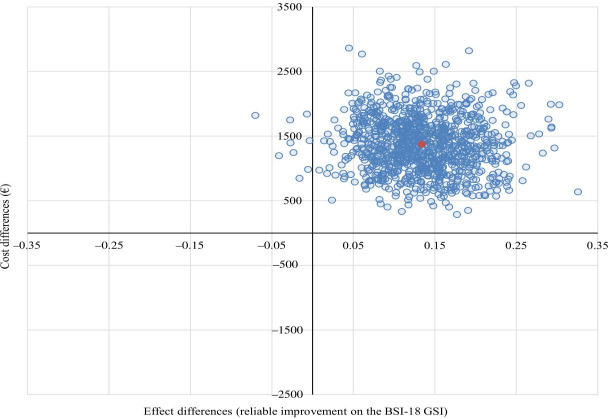
Adjusted cost-effectiveness plane of the i²TransHealth interdisciplinary, internet-based transgender health care program compared with a waiting list for transgender and gender diverse people in northern Germany: analysis from societal perspective with reliable improvement on the BSI-18 GSI as health outcome (cost-differences adjusted for gender identity, age, and total costs at baseline, and effect differences adjusted for gender identity, age, EuroQol 5-dimension 5-level index, and BSI-18 GSI at baseline by seemingly unrelated regression with bootstrapped SEs). A currency exchange rate of €1=US $1.14 was applicable as of December 31, 2020. BSI-18 GSI: Global Severity Index of the Brief Symptom Inventory-18.

The ICER of i²TransHealth per additional reliable improvement on the BSI-18 GSI was €10,058 from an SP. With 99% of the bootstrapped ICERs being in the northeastern quadrant, the cost-effectiveness plane indicated that i²TransHealth was highly likely to be associated with higher costs and additional reliable improvement on the BSI-18 GSI compared with the waiting list (Figure 4). The probability of cost-effectiveness was 0% at a WTP of €0 per additional reliable improvement on the BSI-18 GSI. At a WTP of €5000 and €15,000 per additional reliable improvement on the BSI-18 GSI, the probability of cost-effectiveness was 12% and 75%, respectively.

### Additional Analyses

**Figure 5.**
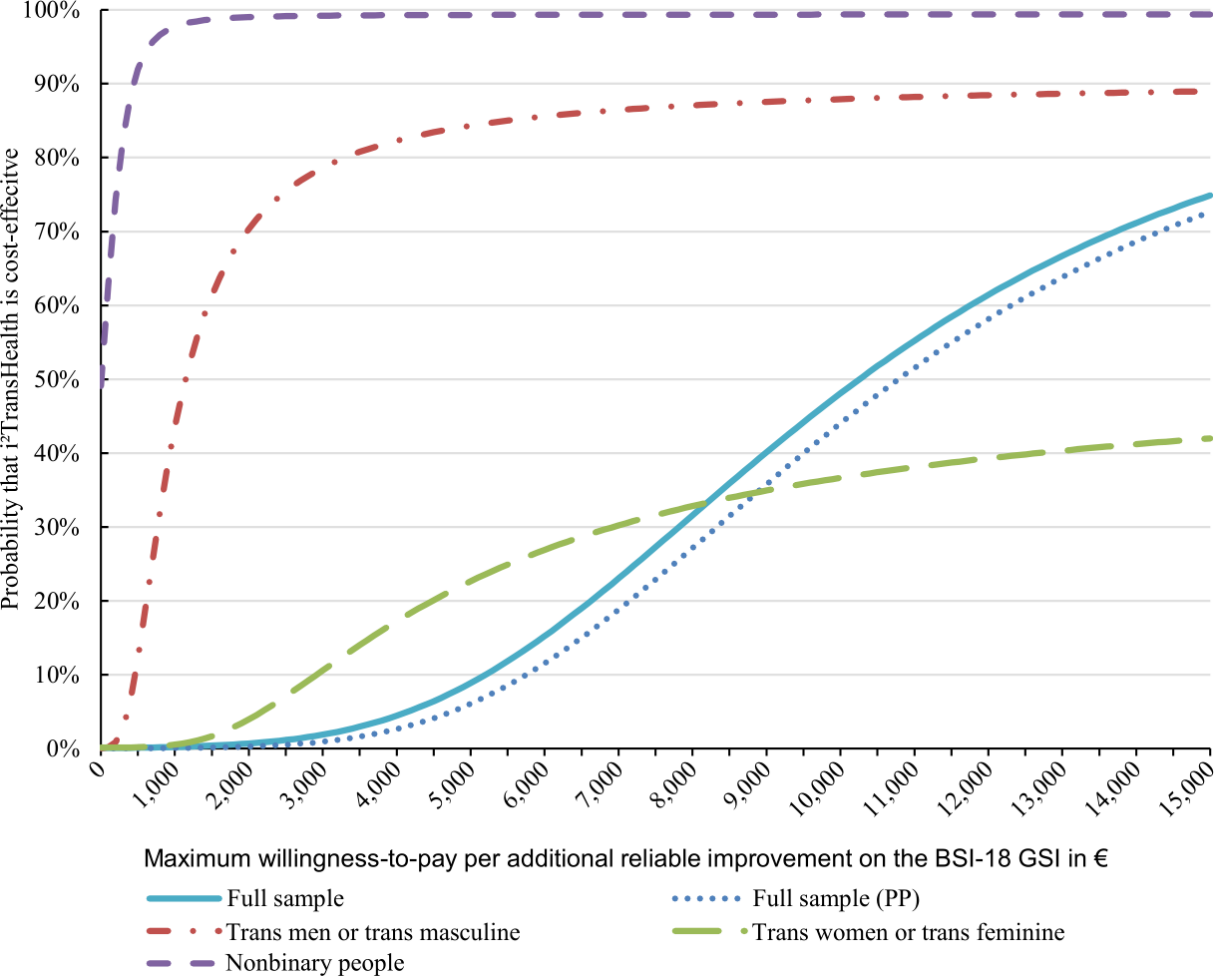
Adjusted cost-effectiveness acceptability curves for an additional reliable improvement on the BSI-18 GSI of the i²TransHealth interdisciplinary, internet-based transgender health care program compared with a waiting list for transgender and gender diverse people in northern Germany: analyses from societal perspective and health care payer’s perspective, and subgroup analysis for trans men, trans women, and nonbinary people from societal perspective (cost-differences adjusted for gender identity, age, and total costs at baseline, and effect differences adjusted for gender identity, age, EuroQol 5-dimension 5-level index and BSI-18 GSI at baseline by seemingly unrelated regression with bootstrapped SEs). A currency exchange rate of €1=US $1.14 was applicable as of December 31, 2020. BSI-18 GSI: Global Severity Index of the Brief Symptom Inventory-18; PP: health care payer’s perspective.

In a subgroup analysis, the probability of cost-effectiveness of i²TransHealth at a WTP of €0 per additional QALY was 0% for trans men and trans women, and 2% and 3% at a WTP of €150,000, respectively. For nonbinary people, however, the probability of cost-effectiveness was 50% at a WTP of €0 and 87% at a WTP of €150,000 (Multimedia Appendix 1). Per additional reliable improvement on the BSI-18 GSI, the probability of cost-effectiveness of i²TransHealth at a WTP of €0 was 0% for trans men and trans women, and 89% and 42% at a WTP of €15,000, respectively. For nonbinary people, the probability of cost-effectiveness was 49% at a WTP of €0 and 99% at a WTP of €15,000 (Figure 5). The differences between participants in the IG and CG in mean total costs, QALYs, and reliable improvement on the BSI-18 GSI, as well as ICERs of i²TransHealth for all analyzed subgroups, are shown in Multimedia Appendix 2.

The additional analysis from a PP using QALYs as an effect measure yielded an ICER of i²TransHealth of €235,896 per additional QALY and a probability of cost-effectiveness of 0% (0% and 17%) at a WTP of €0 (€50,000 and €150,000) per additional QALY (Figure 3). For the effect measure reliable improvement on the BSI-18 GSI, the ICER of i²TransHealth and the probability of cost-effectiveness from a PP was €10,786 and 0% (6% and 73%) at a WTP of €0 (€5000 and €15,000) per additional reliable improvement on the BSI-18 GSI, respectively (Figure 5).

When using QALYs based on the EQ-VAS as an effect measure, the analysis yielded an ICER of i²TransHealth of €1,974,793 per additional QALY. The corresponding probabilities of cost-effectiveness of i²TransHealth at a WTP of €0 per additional QALY were 0% and did not exceed 20% at a WTP of €150,000 per additional QALY based on the EQ-VAS (Figure 3 and Multimedia Appendix 3).

The per-protocol analysis, the analysis with multiply imputed data, as well as the analyses considering winsorized costs and high intervention costs showed ICERs ranging from €170,581 to €274,465 per additional QALY. For the effect measure reliable improvement on the BSI-18 GSI, the ICERs of i²TransHealth ranged from €6915 to €12,078 per additional reliable improvement on the BSI-18 GSI. When considering only mental health–related costs in the analysis, the ICERs were €38,824 per additional QALY and €1427 per additional reliable improvement on the BSI-18 GSI (Multimedia Appendix 4).

## Discussion

### Principal Findings

The aim of this study was to evaluate the cost-effectiveness of the i²TransHealth interdisciplinary, internet-based transgender health care program compared with a waiting list for TGD people from remote areas either exploring their gender identity or being in an early phase of transition from an SP. Overall, i²TransHealth was not cost-effective compared with a waiting list, as a high ICER and low probability of cost-effectiveness were observed even for high WTP. One explanation for this finding might be the inability of the EQ-5D-5L to reflect an improvement in HrQoL relevant to TGD people within a relatively short period of 4 months. Indeed, a statistically significant higher reliable improvement on the BSI-18 GSI associated with GIC or GD was found for participants in the IG. This in turn led to an ICER of around €10,000 and a probability of cost-effectiveness of 75% at a WTP of €15,000 per additional reliable improvement on the BSI-18 GSI. Unlike for an additional QALY, the WTP for an additional reliable improvement on the BSI-18 GSI is unknown, making the results with respect to the probability of cost-effectiveness difficult to interpret. Therefore, the reliable improvement on the BSI-18 GSI was used as an effect measure in addition to QALYs, but no recommendation could be made on this basis.

One further reason for the inability to show the cost-effectiveness of i²TransHealth might be the fact that participants in the CG were placed on a waiting list for 4 months before they were offered an intervention. This relatively short waiting time for transgender health care, which is often significantly longer outside of the study, and the guaranteed usage of either the i²TransHealth intervention or the regular care provided by the Outpatient Unit for Sexual Health and Transgender Care, could have led to a reluctant uptake of health care service usage in general. This would at least partially explain the almost double costs of health care services usage of the participants in the IG compared with the CG during the 4-month follow-up period, with similar costs of health care services usage up to 6 months before baseline.

It is possible that a longer follow-up period would have led to an improvement in HrQoL and a higher improvement on the BSI-18 GSI in the IG. At the same time, there would not have been higher differences in costs of health care services usage between the service users in the IG and CG. This is because the aim of i²TransHealth was to support TGD people in exploring their gender identity and to guide them through the decision-making process with respect to transgender health care but not to initiate transition-related treatments. However, it would not have been ethical to make participants in the CG wait longer for coordinated and integrated transgender health care.

The higher costs of health care services usage of the participants in the IG could also be interpreted as a positive effect of the i²TransHealth interdisciplinary, internet-based transgender health care program. The statistically significant higher costs for medical counseling and transportation among participants in the IG compared with the CG might be an indicator of improved access to transgender health care and a corresponding increase in mobility. However, it is possible that such a positive effect of the i²TransHealth interdisciplinary, internet-based transgender health care program is not equally distributed over subgroups of participants. Furthermore, the costs of the other categories, such as inpatient care and rehabilitation or outpatient physician and mental health specialist services, were not statistically significantly different between the services users in the IG and CG.

i²TransHealth seemed to be rather cost-effective for nonbinary people than for trans men or women, as the probability of cost-effectiveness was already high at a WTP of €50,000 per additional QALY, whereas the probability of cost-effectiveness for trans men and trans women was very low regardless of the WTP. This finding, however, has to be interpreted with caution, as randomization for this study was independent of gender identity, and the analysis was not powered for subgroup analyses. However, as reliable improvement on the BSI-18 GSI associated with GIC and GD was also high among nonbinary participants in the IG and there was no difference in total costs between nonbinary participants in the IG and CG, nonbinary people in particular might benefit from counseling with respect to TGD-informed mental health care and thus from i²TransHealth.

With regard to the further additional analyses, it can be stated that the analysis of the cost-effectiveness of i²TransHealth was robust to alternative approaches and assumptions with respect to the underlying study population, the handling of missing values, and the calculation of total costs.

Even though the additional analyses did not indicate cost-effectiveness from the SP, the i²TransHealth interdisciplinary, internet-based transgender health care program might have improved access to care for TGD people from remote areas either exploring their gender identity or being in an early phase of transition. Furthermore, i²TransHealth was able to reduce the psychological distress associated with GIC and GD at manageable additional costs for the intervention per person within 4 months. Especially for remote areas, the combination of access to an interdisciplinary, internet-based transgender health care program as well as TGD-informed office-based regular care provided by general physicians and psychiatrists when needed might be an easy-to-implement and affordable, yet not proven cost-effective, way to improve transgender health care related to transition. Future studies need to evaluate the cost-effectiveness of the i²TransHealth interdisciplinary, internet-based transgender health care program over a longer period of time compared with a more realistic control condition, such as care as usual or no treatment, instead of a waiting list.

### Strengths and Limitations

With i²TransHealth, the first interdisciplinary, internet-based transgender health care program has been evaluated in the German health care system aiming at TGD people from remote areas either exploring their gender identity or being in an early phase of transition. First, one major strength was the inclusion of such a large sample of people with rare clinical conditions like GIC and GD. Second, overall missing information was so small that an imputation was not necessary. The additional analysis to examine a possible influence of imputation did not change the interpretation of the main results. Third, costs and health effects were compared using seemingly unrelated regressions, allowing to account for baseline imbalances and the correlation of costs and health effects in the analysis of cost-effectiveness [46].

However, some limitations of the study have to be mentioned. Generalizability might be reduced to TGD people from remote areas either exploring their gender identity or being in an early phase of transition in Germany. However, it is expected that the upscaling of i²TransHealth has the potential of directing TGD people from other underserved areas across Europe and the world. The assessment of health care services usage was conducted retrospectively for the period of 6 months at T0 and 4 months at T1. The long retrospective period of 6 months might have made data vulnerable to recall bias. Furthermore, the retrospective assessment for periods of different lengths might have led to over- or underestimation of health care services usage. Study participants could have been asked retrospectively for a shorter period at T0. Furthermore, a follow-up period of 4 months might have been too short to identify an improvement on HrQoL and a further improvement on the BSI-18 GSI. In addition, the short-term time horizon of the study might have excluded future costs of health care services usage with regard to transition-related treatments or avoided future costs of health care services usage with regard to contentment with one’s own gender identity acquired through i²TransHealth.

### Conclusions

The interdisciplinary, internet-based transgender health care program i²TransHealth was unlikely to be cost-effective from the societal perspective, even for a very high WTP per additional QALY. It has to be acknowledged that the comparison of i²TransHealth with a waiting list could have distorted the cost-effectiveness by means of a restrained health care service usage in the CG. As there is no internationally recognized WTP for an additional reliable improvement on the BSI-18 GSI, the probability of cost-effectiveness of i²TransHealth is unclear. Further research is needed in order to explore longer-term effects of i²TransHealth also needed in order to explore longer-term effects of i²TransHealth with regard to possible subsequent transition-related treatments.

## Supplementary material

10.2196/66371 Multimedia Appendix 1 Adjusted cost-effectiveness acceptability curves for an additional quality-adjusted life year of the i²TransHealth interdisciplinary, internet-based transgender health care program compared with a waiting list for transgender and gender diverse people in northern Germany: subgroup analysis from societal perspective with quality-adjusted life years based on the EuroQol 5-dimension 5-level index as health outcome.

10.2196/66371 Multimedia Appendix 2 Adjusted differences between intervention group and control group in mean total costs (from societal perspective), quality-adjusted life years based on the EuroQol 5-dimension 5-level index, reliable improvement on the Global Severity Index of the Brief Symptom Inventory-18 and incremental cost-effectiveness ratio of the i²TransHealth interdisciplinary, internet-based transgender health care program compared with a waiting list for transgender and gender diverse people in northern Germany during 4-month follow-up—subgroup analyses.

10.2196/66371 Multimedia Appendix 3 Adjusted cost-effectiveness plane of the i²TransHealth interdisciplinary, internet-based transgender health care program compared with a waiting list for transgender and gender diverse people in northern Germany: primary analysis from societal perspective with quality-adjusted life years based on the visual analog scale of the EuroQol 5-dimension 5-level index as health outcome.

10.2196/66371 Multimedia Appendix 4 Adjusted differences between intervention group and control group in mean total costs (from societal perspective), quality-adjusted life years based on the EuroQol 5-dimension 5-level index, reliable improvement on the Global Severity Index of the Brief Symptom Inventory-18 and incremental cost-effectiveness ratio of the i²TransHealth interdisciplinary, internet-based transgender health care program compared with a waiting list for transgender and gender diverse people in northern Germany during 4-month follow-up—additional analyses.

10.2196/66371 Checklist 1 Consolidated Health Economic Evaluation Reporting Standards 2022 (CHEERS 2022).

## Abbreviations

BSI-18 GSI: Global Severity Index of the Brief Symptom Inventory-18
CG: control group
CHEERS: Consolidated Health Economic Evaluation Reporting Standards
*DSM-5*: *Diagnostic and Statistical Manual of Mental Disorders, Fifth Edition*
EQ-5D-5L: EuroQol 5-dimension 5-level
EQ-VAS: visual analog scale of the EuroQol 5-dimension 5-level
GD: gender dysphoria
GIC: gender incongruence
HrQoL: health-related quality of life
*ICD-11*: *International Classification of Diseases, 11th Revision*
ICER: incremental cost-effectiveness ratio
IG: intervention group
PP: health care payer’s perspective
QALY: quality-adjusted life year
SP: societal perspective
TGD: transgender and gender diverse
UKE: University Medical Center Hamburg-Eppendorf
WTP: willingness-to-pay
