# Supplementary material for: Cost-Effectiveness of an Interdisciplinary, Internet-Based Transgender Health Care Program in Germany: Economic Evaluation Alongside a Randomized Controlled Trial
Source: J Med Internet Res. 2025 Jun 19;27:e66371. doi: 10.2196/66371 (PMC12202241; doi:10.2196/66371)
Supplement: Multimedia Appendix 1 [file jmir-v27-e66371-s001.docx]

Figure S1. Adjusted^a^ cost-effectiveness acceptability curves for an additional QALY of the i²TransHealth internet-based transgender health care program compared with a waiting list for TGD people in northern Germany: subgroup analysis from societal perspective with QALY-EQ-5D-5L as health outcome.

QALY: quality-adjusted life year

^a^ Cost-differences adjusted for gender identity, age and total costs at baseline, and effect differences adjusted for gender identity, age, EQ-5D-5L index and BSI-18 Global Severity Index at baseline by seemingly unrelated regression with bootstrapped standard errors.
