# Supplementary material for: Cost-Effectiveness of an Interdisciplinary, Internet-Based Transgender Health Care Program in Germany: Economic Evaluation Alongside a Randomized Controlled Trial
Source: J Med Internet Res. 2025 Jun 19;27:e66371. doi: 10.2196/66371 (PMC12202241; doi:10.2196/66371)
Supplement: Multimedia Appendix 2 [file jmir-v27-e66371-s002.docx]

Table S1. Adjusted^a^ differences between intervention group and control group in mean total costs (from societal perspective), QALYs (based on EQ-5D-5L index), reliable improvement on the BSI-18 GSI and ICER of the i²TransHealth internet-based transgender health care program compared with a waiting list for TGD people in northern Germany during 4-month follow up – Subgroup analyses

| **Subgroup** | **n/N (%)** | **Δ total costs** | **Δ QALYs** | **Δ reliable improvement on the BSI-18 GSI** | **ICER per additional QALY** | **ICER per additional reliable improvement on the BSI-18 GSI** |
| --- | --- | --- | --- | --- | --- | --- |
|  |  | **Mean (SE)** | | |  |  |
| **All participants** | 150/168 (89%) | €1390 (€439)*** | 0.01 (0.00) | 13.82%** | €239,118 | €10,058 |
| **Sex assigned at birth** |  |  |  |  |  |  |
| Female | 83/150 (55%) | €1279 (€400)*** | 0.00 (0.00) | 22.53%** | €321,259 | €5677 |
| Male | 67/150 (45%) | €1312 (€815) | 0.01 (0.00) | 4.13% | €262,380 | €31,926 |
| **Gender identity** |  |  |  |  |  |  |
| Trans man/trans masculine | 63/150 (42%) | €1497 (€477)** | −0.00 (0.00) | 14.87% | Dominated | €9986 |
| Trans woman/trans feminine | 50/150 (33%) | €2582 (€912)** | 0.00 (0.01) | 1.93% | €632,313 | €131,967 |
| Non-binary | 37/150 (25%) | −€12 (€939) | 0.01 (0.01) | 30.15%** | Dominant | Dominant |
| **Categorized age** |  |  |  |  |  |  |
| 18 to 24 | 85/150 (57%) | €1102 (€499)* | 0.00 (0.00) | 7.89% | €593,075 | €14,031 |
| 25 to 60 | 65/150 (43%) | €1561 (€765)* | 0.00 (0.00) | 13.57%* | €393,625 | €11,502 |

SE: standard error, QALY: quality-adjusted life year, ICER: incremental cost-effectiveness ratio

^a^ Cost-differences adjusted for gender identity, age and total costs at baseline, and QALY differences/differences in response to treatment adjusted for gender identity, age, EQ-5D-5L index and BSI-18 Global Severity Index at baseline by seemingly unrelated regression with bootstrapped standard errors.

* *P*<.05, ** *P*≤.01, *** *P*≤.001
