# Supplementary material for: Cost-Effectiveness of an Interdisciplinary, Internet-Based Transgender Health Care Program in Germany: Economic Evaluation Alongside a Randomized Controlled Trial
Source: J Med Internet Res. 2025 Jun 19;27:e66371. doi: 10.2196/66371 (PMC12202241; doi:10.2196/66371)
Supplement: Multimedia Appendix 3 [file jmir-v27-e66371-s003.docx]

Figure S2. Adjusted^a^ cost-effectiveness plane of the i²TransHealth internet-based transgender health care program compared with a waiting list for TGD people in northern Germany: primary analysis from societal perspective with QALY-VAS health outcome.

QALY: quality-adjusted life year, VAS: visual analogue scale

^a^ Cost-differences adjusted for gender identity, age and total costs at baseline, and effect differences adjusted for gender identity, age, EQ-VAS and BSI-18 Global Severity Index at baseline by seemingly unrelated regression with bootstrapped standard errors.
