# Supplementary material for: Cost-Effectiveness of an Interdisciplinary, Internet-Based Transgender Health Care Program in Germany: Economic Evaluation Alongside a Randomized Controlled Trial
Source: J Med Internet Res. 2025 Jun 19;27:e66371. doi: 10.2196/66371 (PMC12202241; doi:10.2196/66371)
Supplement: Multimedia Appendix 4 [file jmir-v27-e66371-s004.docx]

Table S2. Adjusted^a^ differences between intervention group and control group in mean total costs (from societal perspective), QALYs (based on EQ-5D-5L index), reliable improvement on the BSI-18 GSI and ICER of the i²TransHealth internet-based transgender health care program compared with a waiting list for TGD people in northern Germany during 4-month follow up – Additional analyses

| **Additional analysis** | **n/N (%)** | **Δ total costs** | **Δ QALYs** | **Δ reliable improvement on the BSI-18 GSI** | **ICER per additional QALY** | **ICER per additional reliable improvement on the BSI-18 GSI** |
| --- | --- | --- | --- | --- | --- | --- |
|  |  | **Mean (SE)** | | |  |  |
| Main analysis | 150/168 (89%) | €1390 (€439) | 0.01 (0.00) | 13.82%** | €239,118 | €10,058 |
| Per protocol analysis | 143/168 (85%) | €1154 (€339)*** | 0.01 (0.00) | 16.69%** | €170,581 | €6915 |
| Analysis with MI data^b^ | 168/168 (100%) | €1171 (€570)*** | 0.00 (0.00) | 11.96%* | €274,465 | €9797 |
| Analysis with winsorized costs^c^ | 150/168 (89%) | €1257 (€297)*** | 0.01 (0.00)* | 14.92%** | €190,101 | €8425 |
| Analysis with only MH costs^d^ | 161/168 (96%) | €203 (€378) | 0.01 (0.00) | 14.24%** | €38,824 | €1427 |
| Analysis with high intervention costs | 150/168 (89%) | €1797 (€421)*** | 0.01 (0.00)* | 14.87%** | €269,151 | €12,078 |

SE: standard error, QALY: quality-adjusted life year, ICER: incremental cost-effectiveness ratio, MH: mental health care, MI: multiple imputation

^a^ Cost-differences adjusted for gender identity, age and total costs at baseline, and QALY differences/differences in improvement of psychological distress adjusted for gender identity, age, EQ-5D-5L index and BSI-18 global severity index at baseline by seemingly unrelated regression with bootstrapped standard errors.

^b^ Data was multiply imputed using predictive mean matching.

^c^ Total costs were winsorized at the 95th percentile.

^d^ Mental health care costs consisted of costs for psychiatric inpatient care, outpatient services by psychiatrists and psychotherapists, psychiatric medication, therapies and counseling.

* *P*<.05, ** *P*≤.01, *** *P*≤.001
