# Supplementary material for: Cost-Effectiveness of an Interdisciplinary, Internet-Based Transgender Health Care Program in Germany: Economic Evaluation Alongside a Randomized Controlled Trial
Source: J Med Internet Res. 2025 Jun 19;27:e66371. doi: 10.2196/66371 (PMC12202241; doi:10.2196/66371)
Supplement: Checklist 1 [file jmir-v27-e66371-s005.pdf]

# CHEERS 2022 Checklist

| | | | |
|---|---|---|---|
| **Title** | | | |
| Title | 1 | Identify the study as an economic evaluation and specify the interventions being compared. | Title, Page 1 |
| **Abstract** | | | |
| Abstract | 2 | Provide a structured summary that highlights context, key methods, results, and alternative analyses. | Abstract, Pages 1-2 |
| **Introduction** | | | |
| Background and objectives | 3 | Give the context for the study, the study question, and its practical relevance for decision making in policy or practice. | Introduction, Fourth Paragraph |
| Methods | | | |
| **Health economic analysis plan** | **4** | **Indicate whether a health economic analysis plan was developed and where available.** | **Not reported** |
| Study population | 5 | Describe characteristics of the study population (such as age range, demographics, socioeconomic, or clinical characteristics). | Methods, Line 10 |
| Setting and location | 6 | Provide relevant contextual information that may influence findings. | Methods (Sample), First and second Paragraph |
| Comparators | 7 | Describe the interventions or strategies being compared and why chosen. | Methods (Interventions) |
| Perspective | 8 | State the perspective(s) adopted by the study and why chosen. | Introduction, Fourth Paragraph |
| Time horizon | 9 | State the time horizon for the study and why appropriate. | Methods (Sample), Fourth Paragraph |

*(continued)*

| | | | |
|---|---|---|---|
| Discount rate | 10 | Report the discount rate(s) and reason chosen. | Not applicable |
| Selection of outcomes | 11 | Describe what outcomes were used as the measure(s) of benefit(s) and harm(s). | Methods (Measures) |
| Measurement of outcomes | 12 | Describe how outcomes used to capture benefit(s) and harm(s) were measured. | Methods (Measures), First and second Paragraph |
| Valuation of outcomes | 13 | Describe the population and methods used to measure and value outcomes. | Methods (Measures), First Paragraph |
| Measurement and valuation of resources and costs | 14 | Describe how costs were valued. | Methods (Calculation of costs) |
| Currency, price date, and conversion | 15 | Report the dates of the estimated resource quantities and unit costs, plus the currency and year of conversion. | Methods (Calculation of costs), First Paragraph |
| Rationale and description of model | 16 | If modelling is used, describe in detail and why used. Report if the model is publicly available and where it can be accessed. | Not applicable |
| Analytics and assumptions | 17 | Describe any methods for analysing or statistically transforming data, any extrapolation methods, and approaches for validating any model used. | Methods (Statistical Analysis) |
| Characterising heterogeneity | 18 | Describe any methods used for estimating how the results of the study vary for subgroups. | Methods (Additional Analyses), First Paragraph |
| Characterising distributional effects | 19 | Describe how impacts are distributed across different individuals or adjustments made to reflect priority populations. | Not applicable |

*(continued)*

| | | | |
|---|---|---|---|
| Characterising uncertainty | 20 | Describe methods to characterise any sources of uncertainty in the analysis. | Methods (Additional Analyses), Second Paragraph |
| Approach to engagement with patients and others affected by the study | 21 | Describe any approaches to engage patients or service recipients, the general public, communities, or stakeholders (such as clinicians or payers) in the design of the study. | Not applicable |
| **Results** | | | |
| Study parameters | 22 | Report all analytic inputs (such as values, ranges, references) including uncertainty or distributional assumptions. | Not applicable |
| Summary of main results | 23 | Report the mean values for the main categories of costs and outcomes of interest and summarise them in the most appropriate overall measure. | Results (Health effects and costs) |
| Effect of uncertainty | 24 | Describe how uncertainty about analytic judgments, inputs, or projections affect findings. Report the effect of choice of discount rate and time horizon, if applicable. | Results (Additional analyses) |
| Effect of engagement with patients and others affected by the study | 25 | Report on any difference patient/service recipient, general public, community, or stakeholder involvement made to the approach or findings of the study | Not reported |
| Discussion | | | |

*(continued)*

| | | | |
|---|---|---|---|
| Study findings, limitations, generalisability, and current knowledge | 26 | Report key findings, limitations, ethical or equity considerations not captured, and how these could affect patients, policy, or practice. | Discussion |
| Other relevant information | | | |
| Source of funding | 27 | Describe how the study was funded and any role of the funder in the identification, design, conduct, and reporting of the analysis | Abstract |
| Conflicts of interest | 28 | Report authors conflicts of interest according to journal or International Committee of Medical Journal Editors requirements. | End of manuscript |

*From:* Husereau D, Drummond M, Augustovski F, et al. Consolidated Health Economic Evaluation Reporting Standards 2022 (CHEERS 2022) Explanation and Elaboration: A Report of the ISPOR CHEERS II Good Practices Task Force. Value Health 2022;25. doi:10.1016/j.jval.2021.10.008
